# Breastfeeding in the time of Zika: a systematic literature review

**DOI:** 10.7717/peerj.6452

**Published:** 2019-02-19

**Authors:** Clara Luz Sampieri, Hilda Montero

**Affiliations:** Instituto de Salud Pública, Universidad Veracruzana, Xalapa, Veracruz, México

**Keywords:** Human-milk, Zika, Transmission, Nursing mother, Human lactation

## Abstract

**Background:**

The disease Zika is considered as emergent. The infection can be acquired through different routes: a bite from the *Aedes* mosquito, sexual contact, from mother to child during pregnancy and by blood transfusion. The possibility of Zika transmission through human lactation has been considered. Zika is a disease of great concern for public health because it has been associated with neonatal and postnatal microcephaly, among other birth defects.

**Objectives:**

To review published evidence of the probable transmission of Zika through human lactation.

**Data sources:**

Electronic databases: Cochrane Central Register of Controlled Trials, EBSCO, Gale, Science Direct, Scopus, US National Library of Medicine (PubMed) and Web of Science. World Health Organization and Centers for Disease Control and Prevention web pages.

**Study eligibility criteria:**

To be eligible, studies of any design had to provide primary data of human breast milk as a potential fluid for the transmission of Zika, or primary or secondary follow-up data of infants with at least one previous published study that complied with the first criterion of eligibility.

**Participants:**

Studies about women with suspected, probable or confirmed Zika during pregnancy, or the postnatal period and beyond. Studies about infants who breastfeed directly from the breast or where fed with the expressed breast milk of the suspected, probable or confirmed women with Zika.

**Results:**

This study only chose data from research papers; no patients were taken directly by the authors. A total of 1,146 were screened and nine studies were included in the qualitative synthesis, from which a total of 10 cases were identified, with documented follow-up in three of these cases. Through the timing of maternal Zika infection, five cases were classified as prenatal (time before delivery), one as immediate postnatal (period from 0 to 4 days after birth); no cases were classified as medium postnatal (period from 5 days to 8 weeks after birth); two were classified as long postnatal (period from 8 weeks to 6 months after birth) and two as beyond six months after birth.

**Conclusion:**

Human milk may be considered as a potentially infectious fluid, but we found no currently documented studies of the long-term complications in infants up to 32 months of age, with suspected, probable or confirmed Zika through human lactation, or evidence with respect to the human pathophysiology of the infection acquired through human lactation. In the light of the studies reviewed here, the World Health Organization recommendation of June 29th 2016, remains valid: “the benefits of breastfeeding for the infant and mother outweigh any potential risk of Zika virus transmission through breast milk.”

## Introduction

Among the diseases that have emerged in the 21st century, Zika is raising some of the greatest concern for public health (Paixao et al., 2016; Wikan & Smith, 2016). Zika virus (ZIKV) infection has been presented in parts of Africa and in South East Asia, and is becoming established in the Americas and Caribbean since its detection in Brazil in 2015 (World Health Organization, 2016a; World Health Organization, 2016b). Infection with ZIKV has usually been associated with asymptomatic or mild illness, known as ZIKV disease, characterized by a usually pruritic and maculopapular rash, with two or more signs or symptoms (fever, non-purulent/hyperemic conjunctivitis, arthralgia, myalgia, peri-articular edema) (Lednicky et al., 2016; World Health Organization, 2016a; World Health Organization, 2016b). In an outbreak in French Polynesia in 2013–2014, there were reports of autoimmune and neurological complications, such as Guillain-Barré syndrome, in the context of co-circulating chikungunya (CHIKV) and dengue (DENV) viruses (Aubry et al., 2015; Musso, Cao-Lormeau & Gubler, 2015).

In 2015, the Brazilian Ministry of Health reported a 20-fold increase in neonatal microcephaly in areas of ZIKV infection outbreak (Gulland, 2016; Schuler-Faccini et al., 2016). Multiple subsequent studies have demonstrated the association of ZIKV infection with neonatal and postnatal microcephaly, other birth defects and Guillain-Barré syndrome (Brasil et al., 2016; Cauchemez et al., 2016; Driggers et al., 2016; Kleber de Oliveira et al., 2016; Chibueze et al., 2017).

The ZIKV is transmitted by the bite of an infected *Aedes* species mosquito, from a pregnant woman to her fetus, from a person who has the disease to his or her sexual partners and through blood transfusion (Centers for Disease Control and Prevention, 2018b; Centers for Disease Control and Prevention, 2018c; Sharma & Lal, 2017). A pioneer report provided possible evidence of peripartum transmission of ZIKV in two neonates in French Polynesia in 2013–2014, although authors even report finding non-replicative ZIKV particles in breast milk, the transmission of ZIKV by breastfeeding was considered (Besnard et al., 2014). The outcome of these two neonates after peripartum acquisition of ZIKV infection at ≈30 months has also been reported (Besnard, Dub & Gerardin, 2017). At this time, the World Health Organization recommended that mothers with suspected, probable or confirmed ZIKV infection, or who reside in or have travelled within 2 weeks to areas of ongoing ZIKV transmission, should start breastfeeding within 1 h of birth, breastfeed exclusively for 6 months and continuing breastfeeding their child up to 2 years of age or beyond (World Health Organization, 2016a). Here, we review evidence of the probable transmission of ZIKV through human lactation and propose some recommendations to improve the translational power of future human studies.

## Research Methods

This study only selected research papers, from which the data were collated. No subjects or clinical samples were taken directly by the authors of this review.

### Search strategy

We searched the following electronic databases: Cochrane Central Register of Controlled Trials (CENTRAL), EBSCO, Gale, Science Direct, Scopus, US National Library of Medicine (PubMed) and Web of Science. Other sources employed were the World Health Organization and Centers for Disease Control and Prevention web pages between the 11th and 16th of July 2018. We designed search terms for each database according to the level of indexation. These search terms were: Zika, Zika virus, breast milk, human milk, breastfeeding, lactation, nursing, lactating women, lactating mother, nursing women and nursing mother (Supplementary material I). There were no restrictions in terms of study design, date or language. We registered this systematic review in PROSPERO, the international prospective register of systematic reviews funded by the National Institute for Health Research and the Centre for Reviews and Dissemination of the University of York, under the number CRD42018102055. The recommendations of the PRISMA group were followed in terms of identification, screening, eligibility and inclusion criteria (Moher et al., 2009).

### Selection of studies

Both authors assessed the titles and abstracts of references of relevance to the objective of this review. For each reference, primary data pertaining to the participants, exposures, outcomes, methods and results were reviewed. The references cited in the studies included were also reviewed. There was no disagreement regarding the eligibility of studies.

### Type of studies included

**Figure 1:**
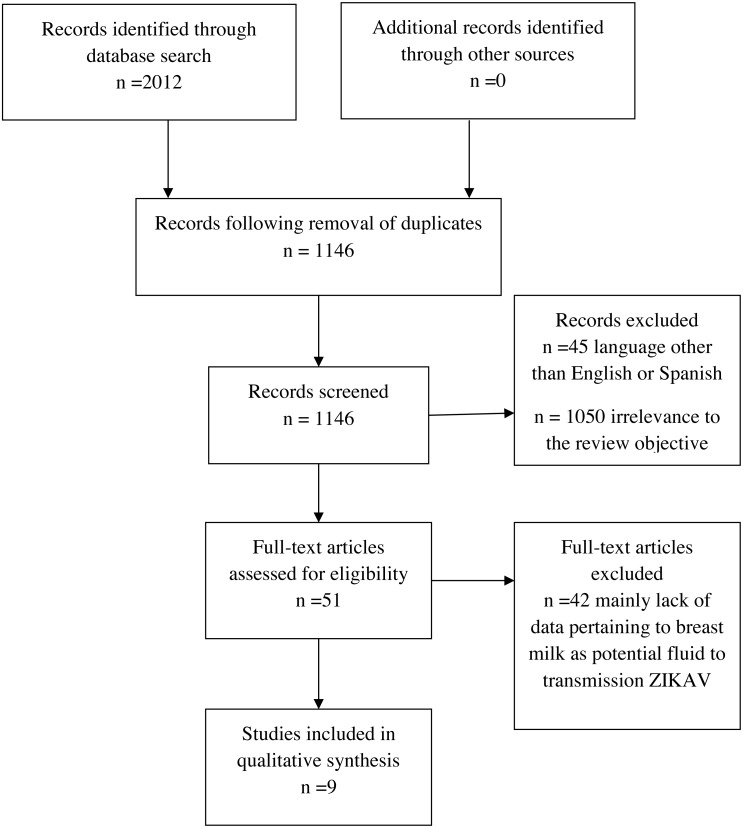
Systematic review process.

We included all studies that comply with the following criteria: (1) studies must comprise primary data produced from any type of study design, (2) participants: mother with infant(s), mother only or infant only, (3) data of breast milk as a potential fluid for the transmission of ZIKV, or (4) primary or secondary data of follow-up of infants with at least one previously published study that complies with criteria 1, 2 or 3 (Table 1). Applying these criteria, nine studies were ultimately included in the qualitative synthesis (Fig. 1).

**Table 1:** Population, intervention, control and outcomes (PICOS model).

Population^a^	Women with suspected, probable or confirmed ZIKAV disease during pregnancy, postnatal period or beyond up to 6 months after the birth of their children, who breastfeed directly from the breast or with someone who feeds the baby with their expressed breast milk.
	Subgroups by timing of maternal ZIKAV disease:
	• I: Prenatal, time before delivery.
	• II: Immediate postnatal, period from 0 to 4 days.
	• III: Medium postnatal, period from 5 days to 8 weeks.
	• IV: Long postnatal, period from 8 weeks to 6 months.
	• V: Period beyond 6 months of age.
	Infant subgroups as:
	• Preterm: <37 completed weeks of gestation.
	• Low birth weight: <2,500 g.
	• Presence of congenital anomaly.
Interventions	1. Breastfeeding directly from the breast at any frequency or for any duration.
	2. Consumption of expressed breast milk in any quantity obtained by any method, alone or in combination (manual, manual pump or electric pump), offered to the infant by any device, alone or in combination (bottle, finger feeding, syringe, tube, supplemental nursing system, cup feeding or spoon).
Control	No breastfeeding directly from the breast and no consumption of expressed breast milk.
Outcomes	Presence of ZIKAV in breast milk.
	Suspected, probable or confirmed ZIKAV infection among infants.

**Notes.**

ZIKAV Zika virus

a According to the World Health Organization classification (World Health Organization, 2016a).

### Data extraction and synthesis

Based on the review objective, we extracted data pertaining to the history of suspected, probable or confirmed maternal Zika disease during pregnancy or the postnatal period, according to the PICOS model. According to the adapted Grading of Recommendations Assessment, Development and Evaluation (GRADE) for observational studies, the overall quality of all proposed outcomes was very low (Balshem et al., 2011; Guyatt et al., 2011; Johnson et al., 2016). All of the corresponding authors of the original studies included in the qualitative synthesis of this review were contacted in order to request additional information regarding the studies.

## Results

Nine published studies were included in the qualitative analysis, eight studies had an observational study design and were original contributions (Besnard et al., 2014; Dupont-Rouzeyrol et al., 2016; Besnard, Dub & Gerardin, 2017; Blohm et al., 2017; Cavalcanti et al., 2017; Sotelo et al., 2017; Blohm et al., 2018; Giovanetti et al., 2018) and one was a secondary source of evidence, a personal communication cited in a systematic review (Colt et al., 2017). In these nine published studies, we identified ten dyads (mother–child pair); for three infants, follow-up was reported. We extracted data regarding country of report, timing of event, isolation of ZIKV by cell culture, genomic and transcriptomic analysis for ZIKV, serological analysis of antibodies against ZIKV, other pathogen tests and clinical analysis of epidemiological encounters (Table 2). Where available, we also extracted information regarding the main clinical characteristics of mother and child, pregnancy-complications, type of delivery, week of gestation at delivery, birthweight, Apgar, postpartum-complications, results of testing of clinical samples, cycle threshold (C_T_), viral load and breastfeeding behavior. Five, one, none, two and two dyads were classified in subgroups I, II, III, IV and V, respectively. (Tables 1, 2 and 3).

**Table 2:** Country of report, timing of event and laboratory research methods utilized in the reviewed studies.

**SG**	**C**	**R**	**Country of report Timing of event**	**Cell lines used to isolate ZIKAV from samples**		**Genomic and transcriptomic analysis for ZIKAV from samples**		**Type of antibodies against ZIKAV from samples**		**Other pathogen results, other analysis performed on the child or epidemiological encounters**
				**Mother**	**Child**	**Mother**	**Child**	**Mother**	**Child**	
I	1	1	French Polynesia December 2013	VERO	NT	Breast milk, saliva and serum by RT-qPCR^a^	Saliva and serum by RT-qPCR^a^	NR	NR	All mother and child samples were negative for DENV
	F1	2	32 months follow-up C1	NA	NA	NA	NA	NR	NR	NR
I	2	3	New Caledonia July 2015	VERO	NT	Breast milk and serum by RT-qPCR^a^	Serum by RT-qPCR^a^	NR	NR	Mother and child blood samples were negative for CHIKV and DENV.
	F2	4	8 months follow-up C2	NA	NA	NA	NA	NR	NR	NA
I	3	5	Brazil NR	NR	NR	Breast milk, serum and urine by RT-qPCR^a^	Serum and urine by RT-qPCR^a^	NR	IgM	NR
I	4	6	Brazil NR	VERO	NR	Blood, urine and breast milk by RT-qPCR^a^	Amniotic fluid, cord blood, placenta and urine by RT-qPCR^a^	NR	NR	Mother had IgM and IgG against DENV, no antibodies against NS1 of DENV were detected and negative for CHIKV
I	5	7	Brazil NR	NR	NR	Serum and breast milk by RT-qPCR^a^ and conventional nested PCR for NS5 Breast milk for viral genomic sequencing	Blood, placenta and urine by RT-qPCR^a^ and conventional nested PCR for NS5 Serum and urine for viral genomic sequencing	NR	NR	Mother had negative result on serological test for toxoplasmosis, CHIKV, DENV, herpes virus 1,2 Mother IgG positive and IgM negative for rubella and cytomegalovirus
II	6	1	French Polynesia February 2014	VERO	NT	Breast milk, serum and urine by RT-qPCR^a^	Serum and urine by RT-qPCR^a^	NR	NR	All mother and child samples were negative for DENV
	F6	2	30 months follow-up C6	NA	NA	NA	NA	NR	NR	Follow-up of liver function/2-250
IV	7	5	Brazil NR	NR	NT	Breast milk, serum and urine by RT-qPCR^a^	Serum and urine NT	NR	NR	Serum and urine samples from the husband during the same period showed no ZIKAV RNA amplification
IV	8	8, 9	Venezuelan March 2016	LLC-MK2 VERO E6	LLC-MK2 VERO E6	Blood by RT-qPCR^b^ Breast milk for viral genomic sequencing	Blood and urine by RT-qPCR^b^ Urine for viral genomic sequencing	IgM IgG	NR	Mother and child had cell cultures negative for CHIKV and DENV1,2,3,4 Mother was negative to parvovirus, cytomegalovirus, Epstein-Barr, varicella zoster and herpes simplex 1,2 viruses
V	9	5	Brazil NR	LLC-MK2 MA-104 VERO	NR	Breast milk, serum and urine by RT-qPCR^a^	Urine by RT-qPCR^a^	NR	NR	A serum sample from her spouse showed ZIKAV no amplification
V	10	5	Brazil NR	NR	NR	Breast milk, serum and urine by RT-qPCR^a^	Serum by RT-qPCR^a^	NR	NR	Child sample was positive for CHIKV

**Notes.**

SG Subgroup by timing of maternal ZIKAV disease ZIKAV Zika virus R reference C case F follow up of case NT No test specified by authors NR Not reported; NA, Not applicable RT-qPCR real time reverse transcription quantitative polymerase chain reaction CHIKV Chikungunya virus DENV Dengue virus

Subgroups by timing of maternal ZIKAV disease.

I Prenatal, time before delivery II Immediate postnatal, period from 0 to 4 days III Medium postnatal, period from 5 days to 8 weeks IV Long postnatal, period from 8 weeks to 6 months V period beyond 6 months

References 1 Besnard et al. (2014) 2 Besnard, Dub & Gerardin (2017) 3 Dupont-Rouzeyrol et al. (2016) 4 Colt et al. (2017) 5 Cavalcanti et al. (2017) 6 Sotelo et al. (2017) 7 Giovanetti et al. (2018) 8 Blohm et al. (2017) 9 Blohm et al. (2018)

In some cases, the real time reverse transcription polymerase chain reaction is not quantitative.

**Table 3:** Results of the reviewed studies.

**SG**	**C**	**R**	**Age of mother (years)**	**Main clinical characteristics**		**Laboratory test for ZIKAV**			**Breastfeeding behavior**
				**Mother**^a^	**Child**^a^	**Mother**^a^	**Breast milk**^a^	**Child**^a^	
I	1	1	Early 30s	Mild rash/−2 to 2 No fever/−2 to 2 Recovered favorably	VD: 38 weeks of gestation Apgar 10-10 Unremarkable/1 to 5 Evolved favorably	Serum^b^ (+) 7.0 × 10^4^/2 Saliva^b^ (+)/2	^b^ (+) 205 × 10^4^/3 Cell culture no replicative particles/3	Serum^b^ (+) 65 × 10^4^/3 Saliva^b^ (+)/3	Breastfeeding began on day of delivery
	F1	2	NA	NA	32 months old Remained asymptomatic Normal neurological development CDAS do not indicate neurocognitive problems	NA	NA		Breastfed for 2 months
I	2	3	27	Fever /0-2 Maculopapular rash/2 Recovered favorably	VD: 37 weeks of gestation Apgar 10 Evolved favorably Asymptomatic	Serum^b^ (+) 3.5 × 10^4^/3	^b^ (+) 85.0 × 10^4^/4 Cell culture infective particles/4	Serum^b^ (A)/3	Breastfeeding began on day of delivery
	F2	4	NA	NA	Personal communication: no long term complications at 8 months of age	NA	NA	NA	NA
I	3	5	33	28 weeks of gestation Macular exanthema on the trunk/1 Exanthema on trunk, arms and legs/2-3 Arthralgia/3-5 Asymptomatic/30 Asymptomatic/postpartum	4 month-old healthy girl No malformation No neuro-ophthalmological findings	Urine^b^ NAM (−)/3-5 Serum^b^ (+) C_T_37.5∕3 − 5 Serum^b^ NAM (−)/30 Urine^b^ (+) C_T_ 38.5/30 Serum^b^ NAM (−)/during 4 months postpartum Urine^b^ NAM (−)/during the first 4 months postpartum	^b^ NAM (−)/during 4 months postpartum	Serum^b^ NAM (−)/during the first 4 months postpartum Urine NAM (−)/during the first 4 months postpartum IgM (−)/during the first 4 months postpartum	Breastfed for 4 months after birth
I	4	6	28	36 weeks of gestation Fever, articular pain, myalgia and rash/−23 After delivery, remained in good clinical condition	D: 38 weeks of gestation Birthweight: 2860 g Apgar 9-10 No evidence of growth restriction, microcephaly, cerebral calcifications After birth, remained in good clinical condition	Blood^b^ (+)/−22 Blood^b^ (−)/−19 Urine^b^ (+)/−19 Blood^b^ (−)/−18 Urine^b^ (+)/−18 Blood^b^ (−)/−9 Urine^b^ (+)/−9	^b^ (+) 244 × 10^4^/−9 Cell culture infective particles/−9 ^b^ (+)/0 ^b^ (+) 21.6 × 10^4^/9 Cell culture infective particles/9	Urine^b^ (−)/0 Placenta^b^ (−)/0 Cord blood^b^ (−)/0 Amniotic fluid^b^ (−)/0	No breastfeeding
I	5	7	32	9 weeks of gestation Diffuse pruritic rash, joint pain	At 22 weeks of gestation ultrasound revealed alteration on left hand, which confirmed at 23 weeks of gestation CD: 38 weeks of gestation Birthweight: 2502 g Severe microcephaly After birth, alteration on left hand is supported by X-ray Possible amniotic band syndrome	^c^ Serum (−)/NR	^b^Prevented detection/2 ^c^ (+)/2 Viral genome sequence	Blood^b^ prevented detection /0 Urine^b^ prevented detection/0 Placenta^b^ (+) C_T_ 33 Urine (+)^c^/1 Serum (+)^c^/1 Urine and serum viral genome sequence Complete genome clustered with 86 bootstrap support with mother strain	NR
II	6	1	Early 40s	Gestational diabetes Intrauterine growth restriction Pregnancy complications Mild fever, rash and myalgia/3 Recovered favorably	CD: 38 weeks of gestation Severe hypotrophy Apgar 8-9-9 Leukopenia and thrombocytopenia/3 Rash and neonatal jaundice/4 Evolved favorably	Serum^b^ (+) 59 × 10^4^/1 Serum^b^ (+) 2.6 × 10^4^/5 Serum^b^ (−)/8 Urine^b^ (+) 16 × 10^4^/8 Serum^b^ (−)/11 Serum^b^ (−)/13	^b^ (+) 2.9 × 10^4^/8 Cell culture no replicative particles/8	Serum ^b^ (−)/0 Serum^b^ (−)/3 Serum^b^ (+) 62 × 10^4^/4 Serum^b^ (+) 69 × 10^4^/7 Urine^b^ (+) 20 × 10^4^/8 Urine^b^ (−)/11	Enteral nutrition with artificial milk began on day of delivery Breastfeeding began on day 3
	F6	2	NA	NA	Approx. 30 months-old Prolonged subclinical hepatitis resolved after 4 months CDAS scores indicated no neurocognitive problems	NA	NA		Breastfed for 6 months
IV	7	5	42	Fever, retro-ocular pain, conjunctival hyperemia, arthralgia rash on face, trunk and members/1-2 Fading exanthema and arthralgia/5	2-3 month-old girl Asymptomatic/5	Serum^b^NAM (−)/5 Urine^b^ (+) C_T_ 36.2/5	^b^ NAM (−)/5	Not tested	Breastfeeding while mother symptomatic
IV	8	8,9	32	Conjunctival hyperemia, pruritic and maculopapular rash/1-5 Malaise, arthralgia/1-11	5 months old Asymptomatic/1-11	Urine^b^ (+) C_T_ 26.73/5 IgM (+)/5 IgG borderline/5	^b^ (+)/5 Cell culture (+) cytopathic effect/5 Viral genome sequence	Plasma^b^ (+) C_T_ 35.57/5 Urine^b^ (+) C_T_ 35.36/5 Urine viral genome sequence Complete genome clustered with 99% bootstrap support with mother strain	Exclusively breastfed
V	9	5	33	Malaise, fever/1 Rash on face, trunk and arms/2 Exanthema both legs/4 Asymptomatic/10 Asymptomatic/17	11 month-old boy Asymptomatic/1 Asymptomatic/3 Asymptomatic/17	Serum^b^ (+) C_T_ 33.4/3 Urine^b^ (+) C_T_ 33.1/3 Serum^b^ NAM (−)/17 Urine^b^ NAM (−)/17	^b^C_T_ (+) 36.7/4 Cell culture infective particles/4 ^b^NAM (−) /17	Urine^b^ (−)/10	Breastfeeding avoided for 7 days after maternal rash and restarted 4 days after maternal rash disappeared
V	10	5	28	1 month of gestation Fever, conjunctival hyperemia, arthralgia and exanthema/1-3 Arthralgia and exanthema relapse/5-6 Rash and arthralgia/7 Asymptomatic/9	10-11 month-old boy Mild infection: fever and exanthema/lasting 2 days No signs or symptoms/5-6 Asymptomatic/7-9	Serum^b^ (+) C_T_38/7 Serum^b^ NAM (−)/9 Urine^b^ (−) C_T_ 38.8/9	^b^NAM (−)/9	Serum^b^ (−)/7	Breastfeeding while mother symptomatic Child serum CHIKV (+)C_T_36.3^b^/7

**Notes.**

SG Subgroup by timing of maternal ZIKAV disease ZIKAV Zika virus R reference C case F follow up of case VD vaginal delivery CD caesarean delivery D delivery CDAS Child development assessment scale NAM No amplification A interpreted as ambiguous result by authors NR Not reported NA Not applicable C_T_ cycle threshold CHIK Chikungunya virus

References 1 Besnard et al. (2014) 2 Besnard, Dub & Gerardin (2017) 3 Dupont-Rouzeyrol et al. (2016) 4 Colt et al. (2017) 5 Cavalcanti et al. (2017) 6 Sotelo et al. (2017) 7 Giovanetti et al. (2018) 8 Blohm et al. (2017) 9 Blohm et al. (2018)

(+) Interpreted as a positive result by authors.

(−) Interpreted as a negative result by authors.

Subgroups according to timing of maternal ZIKAV disease.

I Prenatal, time before delivery II Immediate postnatal, period from 0 to 4 days III Medium postnatal, period from 5 days to 8 weeks IV : Long postnatal, period from 8 weeks to 6 months V period beyond 6 months

a Depending on the case, a negative number after the diagonal symbol means days before delivery, a positive number means days after delivery, where 0 is the day of delivery considering the onset of maternal ZIKAV disease symptoms or mean number of days elapsed since the onset of maternal ZIKAV disease symptoms.

b Real time reverse transcription polymerase chain reaction units: C_T_ or copies/mL (quantitative PCR).

c Nested PCR against NS5 ZIVAV (426 bp).

## Discussion

### Subgroup I

The studies included in this review indicate that five mothers had the onset of symptoms of ZIKV infection prior to the birth of their babies: case 1 (C1), two days before birth (Besnard et al., 2014); C2, on arrival at the hospital for the birth (Dupont-Rouzeyrol et al., 2016); C3, week 28 of gestation (Cavalcanti et al., 2017); C4, week 36 of gestation (Sotelo et al., 2017) and C5, week 9 of gestation (Giovanetti et al., 2018). For this reason, where there was ZIKV infection in the newborns, the probable routes of infection were transplacental or during delivery.

The incubation period for ZIKV, estimated by the Center for Disease Control and Prevention among 197 symptomatic travelers probably infected via mosquito bites, is from 3 to 14 days, with symptoms expected within 1 week of infection in 50% of cases and within 2 weeks in 99% (Krow-Lucal, Biggerstaff & Staples, 2017). This incubation period did not differ qualitatively between pregnant and non-pregnant travellers (Krow-Lucal, Biggerstaff & Staples, 2017). The incubation period in a case of probable ZIKV infection through unprotected sexual intercourse in a pregnant woman at 15 weeks of gestation was reported at 22 days, since ZIKV RNA was detected in plasma and urine samples by real time reverse transcription polymerase chain-reaction (RT-PCR). At delivery, testing was negative for ZIKV in breast milk, umbilical cord, placenta, membranes and serum of the newborn (Desclaux et al., 2018). Moreover, the sequences of NS4 and NS5 ZIKV genes were identical in the plasma of the pregnant woman and semen of her husband (the index case), which is consistent with direct transmission according authors (Desclaux et al., 2018).

In the subgroup I of the reviewed cases (Besnard et al., 2014; Dupont-Rouzeyrol et al., 2016; Cavalcanti et al., 2017; Sotelo et al., 2017; Giovanetti et al., 2018), four newborns were identified born after the full term of pregnancy. For newborn C3, the gestational week at birth was not reported. In three of these cases, the delivery mode is known and in two cases the birthweight is known. Severe congenital abnormalities were reported in newborn C5 only, including microcephaly and alteration on left hand, but it was not specified whether the phase of acute infection by ZIKV in the mother had been confirmed during gestation, moreover, the mother was IgG positive for rubella and cytomegalovirus. The rest of the newborns are referred to as unremarkable or asymptomatic and follow-up is known in two newborns, C1 and C2, to 32 and 8 months, respectively, with both reported as being free of complications (Besnard, Dub & Gerardin, 2017; Colt et al., 2017).

For all of the mothers, except C5, the acute phase of infection by ZIKV was confirmed, with positive results in the serum or blood found using real time reverse transcription quantitative polymerase chain reaction (RT-qPCR) within a period no greater than 3 days from the onset of the symptoms. In mother C5, approximately 29 weeks after the report of symptoms compatible with infection by ZIKV and after delivery of the child, authors report “low viral loads” in the serum that “prevented virus detection by RT-qPCR”, and that the “serum yielded no detectable products” with conventional nested PCR for NS5. In breast milk, they also report that these “prevented virus detection by RT-qPCR”, although there was a positive result using conventional nested PCR for NS5 (Giovanetti et al., 2018).

The presence of ZIKV was confirmed through RT-qPCR and conventional nested PCR for NS5, respectively, in newborns C1 (Besnard et al., 2014) and C5 (Giovanetti et al., 2018) only. For newborn C2, an “ambiguous” result was reported, without specifying the results of other molecular or serological analyses for ZIKV in this child (Dupont-Rouzeyrol et al., 2016). In newborns C3 (Cavalcanti et al., 2017) and C4 (Sotelo et al., 2017), the results for tests for ZIKV disease were reported as negative.

In the breast milk of mothers C1 (Besnard et al., 2014), C2 (Dupont-Rouzeyrol et al., 2016), C4 (Sotelo et al., 2017) and C5 (Giovanetti et al., 2018), the presence of ZIKV was confirmed through RT-qPCR or conventional nested PCR for NS5, with newborns C1 and C2 breastfed from the day of delivery, breastfeeding avoided in newborn C4 and breastfeeding unspecified in newborn C5.

### Subgroup II

Following analysis of the studies included in this review, we classified one case in this subgroup: C6 (Besnard et al., 2014), a mother with ZIKV detected using a RT-qPCR conducted the day after cesarean delivery, with symptoms appearing two days afterwards. The newborn C6 was born at the end of a full term of pregnancy, but its birthweight is not known. Serum testing of the newborn produced a negative result for ZIKV on the first and third days after birth.

Breastfeeding of newborn C6 began on the third day after birth and, on that day, a breast milk sample tested positive for ZIKV by RT-PCR, but no replicative ZIKV were detected in the cell culture. One day after beginning breastfeeding, newborn C6 developed a rash and its serum tested positive for ZIKV by RT-PCR. By day eight after delivery, mother C6 tested negative for ZIKV in serum and positive in urine, her breast milk sample had a positive result for ZIKV, but no replicative ZIKV was detected in cell culture. That same day, newborn C6 tested positive for ZIKV in the serum, but tested negative one day afterwards in the urine.

While there were no results reported for the presence of ZIKV RNA in the placenta, cord blood or amniotic fluid of C6, the authors of the study suggest that the mother “was viraemic or at least incubating ZIKV at the time of delivery”. This interpretation concurs with the incubation period estimated in the travellers (Krow-Lucal, Biggerstaff & Staples, 2017). Moreover, using pooled data of 25 travellers in an endemic area, a mean period of 9.9 days (95% credible interval, 6.9–21.4) was estimated in order to have no detectable ZIKV RNA in the blood following infection, and 95% of cases would be expected to have no detectable virus by 18.9 days (95% CrI: 13.6–79.4) after infection (Lessler et al., 2016). This estimation was made with 11 women, comprising one case of probable sexual transmission, while the rest were presumed to have been infected through mosquito bites, and 14 men, one infected experimentally and the rest presumed to have been infected through mosquito bites (Lessler et al., 2016). It has also been reported in 6 patients that ZIKV RNA was detectable in urine samples at ≤15 days (range 10 days to >20 days) after the onset of symptoms; however, this particular publication did not specify subject sex, age or probable route of transmission (Gourinat et al., 2015).

Considering these estimates of the periods of incubation and viral clearance in the blood, as well as the persistence of ZIKV RNA in urine, it is highly likely that mother C6 acquired the infection by ZIKV during pregnancy, and thus her baby acquired the infection via the transplacental route. The authors state that transmission to newborn C6 via infected mosquito bite “seems fairly improbable because of the air-conditioned rooms in the hospital”, although the temperature inside the hospital was not specified. Newborn C6 evolved favorably, was breastfed for 2 months and, at 32 months, was reported to have no complications (Besnard et al., 2014; Besnard, Dub & Gerardin, 2017).

### Subgroup IV

Following analysis of the studies included in this review, in this subgroup were classified two cases: C7 (Cavalcanti et al., 2017) and C8 (Blohm et al., 2017; Blohm et al., 2018), both mothers with ZIKV infection confirmed in the urine at day five after the onset of symptoms. The delivery mode, birthweight and gestational week at birth of newborns C7 and C8 were not specified.

By day 5, mother C7 had negative results for ZIKV RNA in the serum and breast milk. Her infant “rejected the formula” and continued breastfeeding during the symptomatic phase, and was reported as asymptomatic (Cavalcanti et al., 2017). No studies were carried out to detect ZIKV RNA in the infant, for which reason there is no information with which to determine probable routes of transmission. Moreover, the breast milk, which was the only test conducted, was negative for ZIKV RNA. Over the same period, the husband of mother C7 had negative results for ZIKV RNA in both serum and urine (Cavalcanti et al., 2017).

On day 5 after the onset of symptoms, mother C8 was IgM positive, IgG “borderline” and ZIKV RNA positive in urine, with no results provided for analysis of ZIKV RNA in serum. The authors interpreted the serological data as being indicative of seroconversion. Mother C8 practiced full breastfeeding with her 5 month-old infant, which was reported as asymptomatic throughout the study period (Blohm et al., 2017; Blohm et al., 2018).

The optimal period for IgM detection by enzyme-linked immunosorbent assay (ELISA) and immunofluorescent assay (IFA) has been reported to be from 7 days to 2 months after onset of ZIKV disease (Atif et al., 2016), and for IgG by ELISA, IFA and neutralization test, from 10 days to 6 months after onset of ZIKV disease (Atif et al., 2016). In this sense, it is plausible that mother C8 could have undergone a “rapid seroconversion” due to the physiology of lactation itself.

Specific immunomodulatory constituents of human milk have been reported in maternal human immunodeficiency virus (HIV) infection (Pedersen et al., 2016), maternal breast infections (Hassiotou & Geddes, 2015) and other maternal organ-specific infections (Hassiotou et al., 2013). Moreover, infant infection stimulates a rapid leukocyte response in human milk, where exclusive breastfeeding is associated with a greater baseline level of leukocyte (Hassiotou et al., 2013).

Authors (Blohm et al., 2017) have reported the full genomic sequence of ZIKV RNA, obtained from the spent media of LLC-MK2 cells inoculated with lipid-enriched fraction breast milk of mother C8 and the urine of her child. Both sequences revealed 99% identity between the strains isolated from the mother and child. The authors do not discuss the probable source of infection for mother C8, and there are no references to clinical results in her epidemiological encounters. It is reported that mother C8 and her infant had no recent travel history and lived in Barquisimeto Venezuela in an air-conditioned, screened house, where the infant spent most of its time, without specifying the indoor temperature of the house. The authors (Blohm et al., 2017; Blohm et al., 2018) therefore state that the risk of mosquito transmission to infant C8 would be minimal.

*Aedes aegypti* mosquitoes present a high effectiveness of virus transmission in nature although they tolerate only a narrow range of temperatures (Centers for Disease Control and Prevention, 2018a; Brady et al., 2013; Marinho et al., 2015; Tsai et al., 2018). However, *Aedes albopictus* can tolerate a broader temperature range and cooler temperatures, which enables them to persist in more temperate climates (Brady et al., 2013; Marinho et al., 2015). Moreover, *Aedes albopictus* (Richards et al., 2006) and *Aedes aegypti* mosquitoes are capable of biting more than one person within a household (Chow-Shaffer et al., 2000).

### Subgroup V

Following analysis of the studies included in this review, two cases were classified in this subgroup: C9 (Cavalcanti et al., 2017) and C10 (Cavalcanti et al., 2017), both mothers with ZIKV infection confirmed in serum by real time RT-PCR, but with no specification of delivery mode, birthweight or gestational week of birth of the children.

By day 4 after onset of symptoms, the breast milk of mother C9 was positive for ZIKV RNA and infective particles were detected in the cell culture analysis. Breastfeeding of her infant of 11 months of age was avoided after the onset of the maternal rash and resumed after this maternal rash disappeared. The only test of the infant, conducted in the urine on day 10 after onset of the maternal symptoms, was negative and the infant was reported as asymptomatic at least by day 17 (Cavalcanti et al., 2017).

The breast milk of mother C10 (Cavalcanti et al., 2017), in the only test conducted for detection of ZIKV by day 9 after onset of symptoms, the viral genome was not amplified by real time RT-PCR and her infant of 10–11 months of age, breastfed during the symptomatic maternal period, was found non-reactive for ZIKV, but positive for CHIKV in the only test conducted on the serum. Infant C10 presented cutaneous exanthema and a fever that lasted for 2 days. It should be noted that mother C10 was one month pregnant. Prolonged serum detection of ZIKV RNA in symptomatic pregnant women has been documented at 14 or more days after the onset of symptoms, with a range of 17–46 days (Meaney-Delman et al., 2016).

## Conclusions

Based on the ZIKV RNA detection by real time RT-PCR and the replicative particles found in cell culture, human breast milk may be considered a potentially infectious fluid, although we found no currently documented studies of long-term complications in infants (0–32 months of age). Any change to this situation should be immediately reported, including ZIKV monoinfection and any viral co-infection. While there are reviews of this topic published in 2016 (Grischott et al., 2016), 2017 (Colt et al., 2017; Chibueze et al., 2017; Hagmann, 2017; Sharma & Lal, 2017) and 2018 (Mann et al., 2018), new evidence has recently been reported (Blohm et al., 2018; Giovanetti et al., 2018). In our judgment, the study of Blohm et al. (2018) does not discount that ZIKV infection in the infant could be due to a mosquito bite, moreover ZIKV RNA detection occurs in the urine of the mother and infant at the same time, antibodies are not specified in the infant. On the other hand, in the selection of diagnostic methods for ZIKV we believe that the finding of Giovanetti et al. (2018) in Brazil is of particular interest in endemic zones of the Americas (Giovanetti et al., 2018), since the authors indicate the prevention of virus detection by RT-qPCR, associated with low viral load in breast milk, using the sequences reported by Lanciotti et al. (2008); in our judgment, these sequences have been widely referenced in the literature.

Future studies addressing virus detection, viral load, persistence and frequency of ZIKV in human breast milk could benefit from the inclusion of a consultant or educator in maternal lactation, suitably accredited, for example, by the International Board of Lactation Consultant Examiners or Lactation Education Accreditation and Approval Review Committee (United States Lactation Consultant Association, 2018; Lactation Education Accreditation and Approval Review Committee, 2018). These future studies also need to consider the possibility of co-infection and it would be desirable to classify infants according to consistent breastfeeding definitions (Labbok & Starling, 2012). In conclusion and in light of the reviewed evidence, we consider that the recommendations of the World Health Organization of June 29th, 2016 remain valid: “The benefits of breastfeeding for the infant and mother outweigh any potential risk of Zika virus transmission through breast milk.”

##  Supplemental Information

10.7717/peerj.6452/supp-1 Supplemental Information 1 PRISMA checklist

10.7717/peerj.6452/supp-2 Supplemental Information 2 Supplementary material Records found according to keywords found in a database.

## Additional Information and Declarations

### Data Availability

The following information was supplied regarding data availability: The data are included in the tables.
