# Supplementary material for: Breastfeeding in the time of Zika: a systematic literature review
Source: PeerJ. 2019 Feb 19;7:e6452. doi: 10.7717/peerj.6452 (PMC6385688; doi:10.7717/peerj.6452)
Supplement: Supplemental Information 2 — Records found according to keywords found in a database. [file peerj-07-6452-s002.docx]

| **Cochrane database/ search terms** | **Records found** |
| --- | --- |
| Zika, breast milk | 0 |
| Zika, breastfeeding | 0 |
| Zika, breast feed | 0 |
| Zika, lactating mother | 0 |
| Zika, nursing mother | 0 |
| Zika, lactation | 0 |
| Zika, human milk | 0 |
| Zika, mother milk | 0 |
| **Total records found** | 0 |
| URL  <http://cochranelibrary-wiley.com/cochranelibrary/search> |  |
| Date  15 July 2018 |  |

**Search strategy**

| **EBSCO Database/ search terms** | **Records found** |
| --- | --- |
| Zika, breast milk | 0 |
| Zika, breastfeeding | 537 |
| Zika, breast feed | 0 |
| Zika, lactating mother | 6 |
| Zika, nursing mother | 1 |
| Zika, lactation | 0 |
| Zika, human milk | 6 |
| Zika, mother milk | 1 |
| **Total records found** | 551 |
| **Duplicate records** | 18 |
| **Total records found - duplicate records** | 533 |
| URL  <http://web.a.ebscohost.com.ezproxy.cdigital.uv.mx:2048/ehost/search/basic?vid=0&sid=363f477e-8e28-4bb0-8a97-32c4a742cb82%40sessionmgr4008> | |
| Date  11 July 2018 | |

| **Gale Database/ search terms** | **Records found** |
| --- | --- |
| Zika, breast milk | 1 |
| Zika, breastfeeding | 11 |
| Zika, breast feed | 12 |
| Zika, lactating mother | 6 |
| Zika, nursing mother | 19 |
| Zika, lactation | 14 |
| Zika, human milk | 5 |
| Zika, mother milk | 3 |
| **Total records found** | 71 |
| **Duplicate records** | 15 |
| **Total records found - duplicate records** | 56 |
| URL  http://find.galegroup.com/menu/commonmenu.do?userGroupName=fondoconacyt&pw=redirectme&finalAuth=true | |
| Date  11 July 2018 | |

| **Science Direct database/ search terms** | **Records found** |
| --- | --- |
| Zika, breast milk | 175 |
| Zika, breastfeeding | 123 |
| Zika, breast feed | 145 |
| Zika, lactating mother | 64 |
| Zika, nursing mother | 90 |
| Zika, lactation | 96 |
| Zika, human milk | 369 |
| ZIKA, mother milk | 178 |
| **Total records found** | 1240 |
| **Duplicate records** | 788 |
| **Total records found - duplicate records** | 452 |
| URL  https://www.sciencedirect.com.ezproxy.cdigital.uv.mx:8443/ | |
| Date  12 July 2018 |  |

| **Scopus database/ search terms** | **Records found** |
| --- | --- |
| Zika, breast milk | 28 |
| Zika, breastfeeding | 17 |
| Zika, breast feed | 0 |
| Zika, lactating mother | 1 |
| Zika, nursing mother | 3 |
| Zika, lactation | 6 |
| Zika, human milk | 27 |
| Zika, mother milk | 11 |
| **Total records found** | 93 |
| **Duplicate records** | 52 |
| **Total records found - duplicate records** | 41 |
| URL  https://www-scopus-com.ezproxy.cdigital.uv.mx:8443/search/form.uri?display=basic | |
| Date  15 July 2018 |  |

| **US National Library of Medicine (PubMed)/ search terms** | **Records found** |
| --- | --- |
| Zika, breast milk | 17 |
| Zika, breastfeeding | 16 |
| Zika, breast feed | 10 |
| Zika, lactating mother | 2 |
| Zika, nursing mother | 10 |
| Zika, lactation | 12 |
| Zika, human milk | 10 |
| Zika, mother milk | 8 |
| **Total records found** | 85 |
| **Duplicate records** | 58 |
| **Total records found - duplicate records** | 27 |
| URL  https://www.ncbi.nlm.nih.gov/pubmed/ |  |
| Date  16 July 2018 |  |

| **Web of Science/ search terms** | **Records found** |
| --- | --- |
| Zika, breast milk | 22 |
| Zika, breastfeeding | 13 |
| Zika, breast feed | 1 |
| Zika, lactating mother | 1 |
| Zika, nursing mother | 3 |
| Zika, lactation | 3 |
| Zika, human milk | 10 |
| Zika, mother milk | 9 |
| **Total records found** | 62 |
| **Duplicate records** | 25 |
| **Total records found - duplicate records** | 37 |
| URL  http://apps.webofknowledge.com.ezproxy.cdigital.uv.mx:2048/WOS_GeneralSearch_input.do?product=WOS&search_mode=GeneralSearch&SID=6EMpSJfHim3PgGEqvPw&preferencesSaved= | |
| Date  15 July 2018 | |

| **Centers for Disease Control and Prevention URL reviewed** |
| --- |
| https://www.cdc.gov/zika/index.html |
| Date  10 July 2018 |

| \| **World Health Organization URL reviewed** \| \| --- \| \| http://www.who.int/csr/resources/publications/zika/en/ \| \| http://www.who.int/en/news-room/fact-sheets/detail/infant-and-young-child-feeding \| \| http://www.who.int/csr/resources/publications/zika/breastfeeding/en/ \| \| Date  10 July 2018 \| |
| --- | --- | --- | --- | --- | --- |
